# Late Multiple Organ Surge in Interferon-Regulated Target Genes Characterizes Staphylococcal Enterotoxin B Lethality

**DOI:** 10.1371/journal.pone.0088756

**Published:** 2014-02-13

**Authors:** Gabriela A. Ferreyra, Jason M. Elinoff, Cumhur Y. Demirkale, Matthew F. Starost, Marilyn Buckley, Peter J. Munson, Teresa Krakauer, Robert L. Danner

**Affiliations:** 1 Functional Genomics and Proteomics Facility, Critical Care Medicine Department, Clinical Research Center, National Institutes of Health, Bethesda, Maryland, United States of America; 2 Mathematical and Statistical Computing Laboratory, Center for Information Technology, National Institutes of Health, Bethesda, Maryland, United States of America; 3 Division of Veterinary Resources, Office of Research Services, National Institutes of Health, Bethesda, Maryland, United States of America; 4 Integrated Toxicology Division, U.S. Army Medical Research Institute of Infectious Diseases, Fort Detrick, Maryland, United States of America; Istituto Superiore di Sanità, Italy

## Abstract

**Background:**

Bacterial superantigens are virulence factors that cause toxic shock syndrome. Here, the genome-wide, temporal response of mice to lethal intranasal staphylococcal enterotoxin B (SEB) challenge was investigated in six tissues.

**Results:**

The earliest responses and largest number of affected genes occurred in peripheral blood mononuclear cells (PBMC), spleen, and lung tissues with the highest content of both T-cells and monocyte/macrophages, the direct cellular targets of SEB. In contrast, the response of liver, kidney, and heart was delayed and involved fewer genes, but revealed a dominant genetic program that was seen in all 6 tissues. Many of the 85 uniquely annotated transcripts participating in this shared genomic response have not been previously linked to SEB. Nine of the 85 genes were subsequently confirmed by RT-PCR in every tissue/organ at 24 h. These 85 transcripts, up-regulated in all tissues, annotated to the interferon (IFN)/antiviral-response and included genes belonging to the DNA/RNA sensing system, DNA damage repair, the immunoproteasome, and the ER/metabolic stress-response and apoptosis pathways. Overall, this shared program was identified as a type I and II interferon (IFN)-response and the promoters of these genes were highly enriched for IFN regulatory matrices. Several genes whose secreted products induce the IFN pathway were up-regulated at early time points in PBMCs, spleen, and/or lung. Furthermore, IFN regulatory factors including Irf1, Irf7 and Irf8, and Zbp1, a DNA sensor/transcription factor that can directly elicit an IFN innate immune response, participated in this host-wide SEB signature.

**Conclusion:**

Global gene-expression changes across multiple organs implicated a host-wide IFN-response in SEB-induced death. Therapies aimed at IFN-associated innate immunity may improve outcome in toxic shock syndromes.

## Introduction

Toxic shock [1], [2] overlaps clinically with septic shock [3], [4] and host cytokine release contributes to the pathogenesis of both syndromes. However, unlike septic shock, toxic shock syndrome can occur in the absence of overt infection [1] and the underlying mechanism of immune activation is unique [5], [6], [7]. Toxic shock syndrome toxin 1 (TSST-1) and other exotoxins associated with toxic shock such as staphylococcal enterotoxin B (SEB) are commonly called superantigens. These bacterial toxins bind to the variable region of the T-cell receptor (TCR) beta chain (Vβ) and major histocompatibility complex (MHC) class II molecules on antigen presenting cells (APCs) [5], [8], [9], [10], [11], [12], [13]. The bridging of both cells by superantigen, along with the participation of co-receptors, CD28 on T-cells and CD80 on APCs, leads to massive polyclonal T-cell activation that can result in cytokine storm, shock, organ injury, and death [6], [7], [14]. As humans are extremely sensitive to SEB, especially by inhalation, this superantigen is considered a potential bioterrorism threat [15].

Currently, there are no specific therapies for treating superantigen-induced shock. Low levels of anti-toxin antibody have been identified as a risk factor for severe disease [16], [17], so intravenous immunoglobulin is commonly administered in *de novo* cases of toxic shock syndrome and appears to be beneficial [18], [19]. Inhibiting receptor/toxin interactions has been a major focus of efforts to develop new preventive and/or treatment strategies. These approaches have included vaccines, receptor blocking peptides derived from the toxins, dual-specificity chimeric-inhibitors composed of Vβ and MHC class II domains, and synthetic blockers of the CD28 co-stimulatory receptor [20]. However, specific toxin or receptor inhibitors may lack effectiveness against all potential agents and are likely immunogenic. Furthermore, strategies that seek to prevent the first step in T-cell and APC co-activation may be ineffective in post-exposure treatment [20]. Patients with *de novo* toxic shock are generally diagnosed after the onset of cytokine storm, as would be the case for casualties from an act of bioterrorism. Targeting host-responses downstream from superantigen exposure is therefore appealing clinically. However, some cytokines linked to toxic shock pathogenesis, such as TNFα and IL-1β, are released hyper-acutely [6], [21], [22]. By analogy, targeting these and similar mediators in septic shock syndrome failed to improve survival [23]. Candidate host responses that are both slow to develop and central to outcome have been elusive.

An obvious step in developing new therapeutic approaches for SEB-induced toxic shock is finding relevant models that mimic important aspects of human disease. Compared to humans, mice are much less susceptible to SEB due to its decreased affinity for mouse MHC class II molecules [24], [25], [26]. Therefore, mouse models of SEB-induced shock have used potentiating agents such as lipopolysaccharide [27], viruses [28], D-galactosamine [29], or actinomycin D [30] to amplify the toxic effects of SEB. However, the sensitizing agents themselves often activate similar cell populations *in vivo* through different signaling pathways, and therefore, potentially interact with the effects of SEB in unpredictable ways [31], [32]. To overcome these limitations, a “double-hit” low dose SEB model was developed in C3H/HeJ mice, a lipopolysaccharide (LPS) resistant mouse strain with defective Toll-like receptor 4 (TLR-4) signaling, to investigate the pathological consequences of SEB-induced shock without the use of synergistic agents [31]. This “SEB-only” toxic shock model relies on the intranasal (*i.n.*) administration of SEB followed by a second intraperitoneal (*i.p.*) dose 2 hours later. These two relatively small SEB challenges trigger intense inflammation in the lung and systemic cytokine release that culminate in death more than 3 days after the initial exposure. Importantly, cytokine release, pathological lesions, and time to lethality in C3H/HeJ mice resemble findings in primate studies [30], [33], [34], [35] and clinical staphylococcal toxic shock syndrome in patients [1]. Furthermore, respiratory system challenge with the induction of a pulmonary inflammatory response simulates the manner in which SEB would be used as a bioweapon [15].

In this study, oligonucleotide microarrays were used to examine the temporal pattern of SEB-induced gene expression in peripheral blood mononuclear cells (PBMC), spleen, lung, liver, kidney, and heart to identify common molecules and pathways that might serve as post-exposure therapeutic targets. The extent to which SEB responses, independent of synergistic agents, are distinct or similar across different organs at the transcriptional level has not been previously investigated. The murine toxic shock model employed here does not require high doses of SEB or second agents to produce lethality [31]. Furthermore, mice become moribund relatively late, better reflecting the time course of human toxic shock syndromes.

## Materials and Methods

### Mouse model of SEB-mediated shock

The Institutional Animal Care and Use Committee (IACUC) of the U.S. Army Medical Research Institute of Infectious Diseases (USAMRIID) approved the protocols, AP06-063 and AP10-002, under which this study was conducted. All reported research was performed in compliance with the Animal Welfare Act and other federal statutes and regulations relating to experiments involving animals and adhered to the principles in the Guide for the Care and Use of Laboratory Animals, National Research Council, 1996. This research was conducted in a facility accredited by the Association for Assessment and Accreditation of Laboratory Animal Care International.

Male C3H/HeJ mice (National Cancer Institute, Frederick, MD), weighing ∼20 g each (7–10 weeks old), were housed in conventional microisolator cages. Food and water were freely available at all times. Purified SEB was procured from Toxin Technology (Sarasota, FL) and diluted in sterile, endotoxin-free phosphate-buffered saline (PBS, Sigma-Aldrich, St. Louis, MO). Frozen (−70°C) aliquots of toxin were used for all subsequent studies. SEB was administered *i.n.* (5 µg/dose/mouse in 50 µl) with a micropipet and *i.p.* (2 µg/dose/mouse in 200 µl) with a tuberculin syringe (26G–3/8 inch needle), given 2 h apart. This was the optimal timing and dose previously determined to cause shock and death without the use of synergistic agents [31]. Control C3H/HeJ mice were given two doses of saline (*i.n.* and *i.p.*) 2 h apart similar to the SEB-exposed mice. An intramuscular-injected mixture of ketamine (2.4 mg/kg), acepromazine (0.024 mg/kg), and xylazine (0.27 mg/kg) was used to anesthetize mice prior to all *i.n.* challenges. Mice exposed to both doses of SEB succumb to death between 72 and 120 h.

Blood was collected into EDTA-treated tubes from anesthetized and subsequently euthanized mice by cardiac puncture at 2.75 h, 5 h, and 24 h after *i.n.* SEB. Blood was then immediately diluted with an equal volume of PBS and layered over Nycoprep 1.077 (Sigma-Aldrich). PBMCs were collected at the interface after centrifugation at 700 g for 30 min. Organs were excised from matched, euthanized animals at the same times post-challenge as blood samples. Tissue samples for microarray were thinly sliced and preserved in RNAlater Stabilization Reagent (Qiagen, Valencia, CA) and stored at −80°C.

### Total RNA isolation and microarrays

PBMCs were lysed with RLT buffer (Qiagen) and homogenized (Qiashredder column). Total RNA was extracted using RNeasy mini kits (Qiagen), following the manufacturer's instructions. Organ samples were disrupted utilizing a TissueLyser (Qiagen), and total RNA was isolated using RNeasy mini kits (Qiagen). The quality of total RNA was evaluated using RNA 6000 Nano LabChips (Agilent 2100 Bioanalyzer, Santa Clara, CA). All samples had intact 18S and 28S ribosomal RNA bands with RNA integrity numbers (RIN) between 7.1 to 9.4, and RNA A_260/280_ ratios between 1.9 and 2.0.

Double-stranded cDNA was synthesized from total RNA (2 µg) using GeneChip Expression 3′-Amplification Reagents One-Cycle cDNA Synthesis kits (Affymetrix, Santa Clara, CA, USA). Purified cDNA was then used for *in vitro* synthesis of biotin labeled cRNA (IVT GeneChip Expression 3′-amplification kits; Affymetrix). Labeled cRNA was purified and fragmented (20 µg) for 35 min at 95°C in fragmentation buffer (200 mM Tris-acetate, pH 8.1, 500 mM potassium acetate, 150 mM magnesium acetate). Fragmented cRNA was evaluated using Flash Gel System (Lonza, Walkersville, MD) after which fragmented cRNA (15 µg) was hybridized to Mouse Genome 430 2.0 arrays (Affymetrix) for 16 h at 45°C. Arrays were washed and stained using GeneChip Hybridization, Wash, and Stain kit (Affymetrix) on the Affymetrix Fluidics Station 400. Microarrays were then scanned in an Affymetrix 7G scanner.

### Microarray analysis

After scanning, CEL files were transferred to the NIHAGCC database for archival storage and later analyzed using Affymetrix Expression Console and the MAS5 algorithm for the Mouse 430_2 microarray. Resulting Signal Intensity values and Present-Absent calls for each of 45,101 probesets were processed in JMP statistical package (SAS, Cary, NC) using the Mathematical and Statistical Computing Laboratory (MSCL) Analyst's Toolbox [36], written by and freely available (http://abs.cit.nih.gov/MSCLtoolbox/) from one of the authors (PJM). The quantile-normalizing, variance-stabilizing “S10” transform was applied to the data, separately for each tissue (PBMC, spleen, lung, liver, kidney, and heart). Separate normalization by tissue was performed because gene expression was expected to be widely divergent across different tissues. Data for each tissue was subjected to principal components analysis (PCA). Possible outlier microarrays were first identified by PCA plot inspection; quality control parameters of potential outliers (cRNA yield, percent present, raw Q [noise], scaling factor actin 3′/5′ ratio) were examined to further adjudicate possible censure. The microarray data used in our analysis including CEL files has been deposited in the Gene Expression Omnibus Database (GEO) (http://www.ncbi.nlm.nih.gov/gds) and is available under accession number GSE52474.

After removal of outliers, 154 of 168 microarrays were analyzed using a one-way, seven level ANOVA, with levels C0, C2, C5, C24, S2, S5 and S24, corresponding to baseline animals (C0), and controls at 2.75, 5 and 24 h, and SEB challenged animals at 2.75, 5 and 24 h. Each level had at least 3 biological replicates for each tissue and time point. Selection of probesets as differentially expressed in any individual tissue required a false discovery rate (FDR) of less than or equal to 5%, fold-change ≥1.5 between SEB-challenged and control samples at one or more time points, and a present call in ≥50% of samples at any level. Requiring that these criteria were met across all six tissues retained 103 probesets that annotated to 85 unique gene identifications. Because these transcripts were subsequently recognized as interferon (IFN) response genes (see below), a list of 26 secreted IFN pathway initiators was constructed from a search of PubMed and other online sources. Of these, 19 mapped to probesets on our mouse microarrays and 8 were differentially regulated (FDR ≤5%, fold-change ≥1.5 and present call ≥50% at any level) in at least one tissue and time point.

In a separate analysis to test for the effects of time, stress and anesthetic agent use in the absence of SEB challenge, baseline animals at time 0 were compared to controls at 2.75, 5 and 24 h, applying the same selection criteria used in our primary analysis. Within the controls, no differentially expressed genes were found for spleen, liver, kidney and heart, while PBMC and lung returned only 3 transcripts each. Furthermore, the 6 transcripts that changed significantly over time under control conditions in PBMC and lung showed no overlap with the SEB-induced gene signature reported here.

### Quantitative real-time PCR (qRT-PCR)

High Capacity cDNA Reverse Transcription kits and gene specific primers and probes for Cxcl11 (cat # mm00444662_m1), Herc6 (cat # mm0134963_m1), Irf1 (cat # mm01288580_m1), Irf8 (cat # mm00492567_m1), Irgm1 (cat # mm00492596_m1), Parp12 (cat # mm00556509_m1), Stat1 (cat # mm00439518_m1), Xaf1 (cat # mm00776505_m1), and Zbp1 (cat # mm00457979_m1) were purchased from Applied Biosystems (Foster City, CA). Transcript levels were then measured using TaqManH Universal PCR master mix, and the ABI Prism 7900 sequence detection system (Applied Biosystems). GAPDH was significantly affected by SEB challenge, precluding its use as a housekeeping gene to normalize expression [37]. Fold-change from control measured by qRT-PCR was compared directly with microarray results for the same target gene. Because microarrays tend to underestimate the magnitude of differential expression, only the probeset with the largest fold-change is shown for target genes with multiple retained probesets. Thematic analysis.

Probesets differentially expressed across all tissues were uploaded into Ingenuity Pathway Analysis (IPA®) and examined using the Bio Function, Canonical Pathway and Upstream Regulator applications. Significant upstream regulators were chosen to construct a network of gene interactions based on the known molecular mechanism of cellular activation by SEB and the predominant signature for IFN-regulated genes. These included the T-cell receptor (TCR), IFN pathway initiators expressed early in PBMCs, spleen and lung (TNF, IL-1β, IL-2, IFNγ and IL-12B), and any upstream regulator differentially expressed across all tissues. The resulting network connected 70 of 79 genes recognized by IPA®. The remaining 9 genes and their connections were manually curated using published articles [38], [39], [40] from PubMed (http://www.ncbi.nlm.nih.gov/pubmed/) searches and STRING version 9.05 (http://string-db.org/newstring_cgi/show_input_page.pl?UserId=vYMVCKsJvLc4&sessionId), a database of known and predicted protein-protein interactions [41].

The apparent enrichment of IFN-regulated genes among our transcripts differentially regulated across all tissues was further explored using Interferome v2.01 (http://interferome.its.monash.edu.au/interferome/). This database contains a comprehensive list of IFN-regulated genes manually curated from publicly available microarray datasets, as well as tools for analyzing IFN-regulated gene signatures in experimental results [42]. Genes regulated across all tissues were examined for evidence of an IFN-regulated gene signature. IFN subtype, concentration and timing were unrestricted (default settings), as were biological system and cell-type. Both *in vitro* and *in viv*o experimental results were permitted, but the analysis was restricted to mouse data, corresponding to our animal model. The database recognized 85 uniquely annotated genes from the all-tissue list as IFN-regulated genes, which were then classified by IFN subtype.

### Promoter analysis

The promoters of genes regulated across all tissues were initially analyzed within Interferome v2.01 for IFN pathway-driven regulatory binding sites [42]. This tool relies on TRANSFAC® Professional (2012) matrices and the MATCH™ algorithm with settings to minimize false positives. Next, ExPlain 3.1 (BIOBASE Knowledge Library: http://www.biobase-international.com/; Beverly, MA) was used to determine if IFN-driven transcription factor binding sites were truly over-represented relative to other matrices. In the F-Match module, 492 mouse housekeeping gene promoters were used as the “No-set”. The “vertebrate_all,” minimize false positives profile of position weight matrices was chosen to identify the most promising potential binding sites. Using only the best-supported promoters (n = 84) from our all-tissue list of differentially expressed genes, the maximum promoter window was set at −500 to +100 bp, with cut-off and window optimization, and a p-value threshold of 0.01.

### Histopathology and immunohistochemistry

Organs were excised from euthanized control and SEB-exposed C3H/HeJ mice at the indicated times and emersion-fixed in 10% neutral-buffered formalin. Formalin fixed specimens were embedded in paraffin before sectioning at 5 micron and mounting on glass slides. Slides were deparaffinized with xylene, hydrated through graded alcohols to water and stained with hematoxylin and eosin. Stained samples were dehydrated through graded alcohols to xylene and sealed using Permount™.

Only lung tissue showed characteristic SEB-associated histopathological changes at 24 h post-SEB exposure, the last time point corresponding to our microarray analysis and was subjected to further analysis. For TdT-mediated dUTP-biotin nick end-labeling (TUNEL) assay, sections were deparaffinized with xylene, hydrated and pretreated with proteinase K followed by EDTA and BSA blocking. Samples were then stained with anti-DIG 1∶1000 (Roche Applied Science, USA; cat # 1093274) and fuchsin and counterstained with hematoxylin followed by dehydration through graded alcohols to xylene prior to sealing with Permount™. For immunohistochemistry, sections were deparaffinized and heated in a steamer at 90°C for 20 min to retrieve antigen. Sections were blocked with hydrogen peroxide and BSA, and then washed with Tris-Buffered Saline and Tween 20 (TBST). This was followed by overnight incubation at room temperature with primary antibody, either rabbit polyclonal anti-nitrotyrosine (Abcam, Cambridge, MA; cat # ab42789) or anti-polyADP-ribose (BD Bioscience, San Jose, CA; cat # 551813), each diluted 1∶200. Slides were subsequently washed with TBST and incubated with secondary goat anti-rabbit antibody (Vector Laboratories, Burlingame, CA; cat # BA-1000) diluted 1∶500 for 30 min followed by streptavidin-HRP 1∶400 for 30 min, both at room temperature. Finally, slides were developed with DAB (diaminobenzidine tetrahydrochloride) and counterstained with Carazzi's hematoxylin.

Histopathology and TUNEL assays were initially read by one author (MFS) blinded to challenge and time point. Lung tissue was available from three control and three SEB-challenged animals at 24 h, and one control and two SEB-challenged animals at 48 h. For TUNEL analysis, lung sections were macroscopically divided into five regions within which five high-powered fields (HPFs) were examined in each for a total of 25 HPFs per slide. The number of apoptotic cells in each HPF was quantitated and overall results were subjected to a one-way analysis of variance (ANOVA) combining the four controls, followed by post-hoc contrasts using unpaired t-tests and Bonferroni corrections.

## Results

### SEB-induced a shared genomic response in PBMCs and all organs

Over time in control animals, no transcripts significantly changed in spleen, liver, kidney and heart, and only three each were altered in PBMC and lung (data not shown). Importantly, none of these time-affected transcripts in controls overlapped with the SEB gene signature described below.

SEB challenge compared to control had its earliest and strongest effects in PBMCs (Tables 1 and 2). Responses in spleen and lung, and finally liver, kidney, and heart were progressively delayed (Table 1), involved fewer transcripts, and became increasingly biased toward gene induction compared to suppression (Table 2). However, the number of affected transcripts in each tissue continued to increase with time and some met criteria for differential regulation (≤5% FDR; ≥1.5-fold-change compared to control; and ≥50% present call) in all tissues. These latter transcripts were uniformly induced and accounted for a substantial proportion of all differentially regulated probesets (>30%) in liver, kidney and heart (Table 2). See Table S1 for a complete organ-by-organ list of differentially expressed transcripts.

**Table 1:** Tissue and time specific counts of differentially expressed probesets shown at each time point.

	Time = 2.75		Time = 5		Time = 24	
Tissue	Down	Up	Down	Up	Down	Up
**PBMC**	193	119	1017	843	1843	1437
**Spleen**	3	36	10	204	378	460
**Lung**	11	10	8	172	275	632
**Liver**	3	4	7	21	23	229
**Kidney**	0	0	3	19	20	315
**Heart**	3	0	4	11	12	262
**All** a	0	0	0	1^b^	0	101

a Irf8 (probeset ID 1416714_at) met selection criteria at 5h in PBMC and spleen, and at 24 h in lung, liver, heart, and kidney. Cxcl9 (probeset ID 1456907_at) met selection criteria at 5 h in PBMC, spleen, lung, and kidney, and at 24 h in spleen, lung, liver, kidney, and heart. Therefore, these probesets are not counted in all-tissue totals at specific time points.

b Tgtp1//2 (probeset ID 1449009_at) met all-tissue criteria at both 5 h and 24 h and, therefore, is counted in this table at both time points.

**Table 2:** Tissue specific counts of differentially expressed probesets aggregated across time points.

	Across All Times^a^				
Tissue	Down	Up	Down & Up	Total	% All Tissues
**PBMC**	2219	1905	116	4008	3%
**Spleen**	384	574	8	950	11%
**Lung**	290	669	10	949	11%
**Liver**	32	243	9	266	39%
**Kidney**	23	315	3	335	31%
**Heart**	18	267	5	280	37%
**All**	0	103	0	103	100%

a In each individual tissue, some probesets met selection criteria for down-regulation at one time point and up-regulation at another. Row totals count each probeset once using the following formula: (Down) + (Up) – (Down & Up).

Focusing on the shared SEB-response from Tables 1 and 2, 103 probesets representing 85 uniquely annotated transcripts (Table 3) were identified as differentially expressed across all six tissues. Notably, six transcripts induced in all tissues had maximal changes in excess of 100-fold and somewhat unexpectedly, these large effects were seen in heart (Igtp), kidney (Iigp1, Gbp6, and Gbp6/10), liver (Tgtp1/2), and lung (Cxcl9) rather than tissues primarily composed of lymphocytes (PBMCs and spleen). Many of the genes significantly induced in all tissues have not been previously associated with SEB responses. SEB challenge affected DNA/RNA sensors such as Ifih1 (Mda5) and Zbp1; IFN-induced genes (Ifit1, Ifit2, Ifit3, Ifi47, Ifi202b, Igtp, Iigp1 Iigp2, Stat1, Irf1, Irf7, and Irf8) including those with direct antiviral activity (Dhx58, Herc6, Isg15, Oas1a, Oasl2, Samhd1); apoptosis/DNA damage-related molecules (Dtx3l, Parp9, Parp12, Parp14, Tnfsf10, and Xaf1); signal transduction effectors (Gbp1, Gbp2, Tgtp, and Irgm1); innate inflammatory response mediators and regulators (Cxcl9, Cxcl10, Cxcl11, Il18bp, and Trafd1); cell receptors (Cd274, H2T10, Fcgr4, Ly6a, Ly6c, Ly6e); immunoproteasome components (Psme2, Psmb8, Psmb9, and Psmb10); and ER/metabolic stress pathway genes (Eif2ak2, Erap1, and Ubd). Note these functional classifications are offered as examples and are neither complete nor mutually exclusive. Overall, inspection of the 85 transcripts that were uniformly up-regulated across all tissues (Table 3), suggested that lethal SEB challenge in this model triggered a widespread genetic program characterized by IFN, stress and damage pathway responses.

**Table 3:** Eighty-five annotated genes were up-regulated in all six tissues.

Gene Symbol	Gene Title	Entrez Gene ID	Tissue and Time (hours) of Maximum Fold Change	Maximum Fold Change
Igtp	interferon gamma induced GTPase	16145	Heart 24	168.88
Iigp1	interferon inducible GTPase 1	60440	Kidney 24	132.73
Tgtp1; Tgtp2	T cell specific GTPase 1; T cell specific GTPase 2	21822; 100039796	Liver 24	127.34
Gbp6^a^	guanylate binding protein 6	100702	Kidney 24	118.55
Gbp6; Gbp10^a^	guanylate binding protein 6; guanylate-binding protein 10	100702; 626578	Kidney 24	114.21
Cxcl9	chemokine (C-X-C motif) ligand 9	17329	Lung 24	103.17
Irgm2	immunity-related GTPase family M member 2	54396	Heart 24	49.79
Cxcl10	chemokine (C-X-C motif) ligand 10	15945	PBMC 5	49.25
Gbp1; LOC100047734	guanylate binding protein 1 (interferon-induced)	14468	Kidney 24	45.03
Gbp2	guanylate binding protein 2	14469	Kidney 24	39.18
Gbp8	guanylate-binding protein 8	76074	PBMC 24	35.59
Ly6a	lymphocyte antigen 6 complex, locus A	110454	PBMC 24	34.74
Psmb8	proteasome subunit, beta type 8 (large multifunctional peptidase 7)	16913	Kidney 24	27.10
Irgm1	immunity-related GTPase family M member 1	15944	Heart 24	27.05
Cd274	CD274 antigen	60533	Heart 24	25.27
Stat1	signal transducer and activator of transcription 1	20846	Kidney 24	24.37
Ubd	ubiquitin D	24108	Kidney 24	20.07
Serpina3g	serine (or cysteine) peptidase inhibitor, clade A, member 3G	20715	Lung 24	18.66
Psmb9	proteasome subunit, beta type 9 (large multifunctional peptidase 2)	16912	Heart 24	17.99
Fam26f	family with sequence similarity 26, member F	215900	Lung 24	17.39
Gbp3	guanylate binding protein 3	55932	Kidney 24	16.91
Ifi47	interferon gamma inducible protein 47	15953	Kidney 24	16.01
Psmb10	proteasome (prosome, macropain) subunit, beta type 10	19171	Kidney 24	15.49
Gbp7	guanylate binding protein 7	229900	Heart 24	14.91
Cxcl11	chemokine (C-X-C motif) ligand 11	56066	Lung 24	14.07
Ifit1	interferon-induced protein with tetratricopeptide repeats 1	15957	PBMC 24	13.98
Oasl2	2′-5′ oligoadenylate synthetase-like 2	23962	Spleen 24	13.11
H2-T23; C920025E04Rik	histocompatibility 2, T region locus 23; RIKEN cDNA C920025E04	15040; 667803	Heart 24	12.89
Il18bp	interleukin 18 binding protein	16068	PBMC 24	12.63
Zbp1	Z-DNA binding protein 1	58203	Lung 24	12.00
Isg15; Gm9706^b^	ISG15 ubiquitin-like modifier; predicted gene 9706	100038882; 677168	PBMC 24	11.68
Ifi202b; LOC100044068^c^	interferon activated gene 202B; interferon-activable protein 202-like	26388; 100044068	Lung 24	11.68
Ifit3	interferon-induced protein with tetratricopeptide repeats 3	15959	Heart 24	11.02
Ifi202b^c^	interferon activated gene 202B	26388	Lung 24	10.63
Usp18	ubiquitin specific peptidase 18	24110	PBMC 24	10.34
Ifi44	interferon-induced protein 44	99899	Lung 24	9.56
Ifit2	interferon-induced protein with tetratricopeptide repeats 2	15958	Lung 24	9.50
AW112010	expressed sequence AW112010	107350	Lung 24	9.11
Irf7	interferon regulatory factor 7	54123	Heart 24	8.40
Gvin1; Gm4070	GTPase, very large interferon inducible 1; predicted gene 4070	74558; 100042856	Kidney 24	8.27
Fcgr4	Fc receptor, IgG, low affinity IV	246256	PBMC 24	8.03
Wars	tryptophanyl-tRNA synthetase	22375	Heart 24	7.78
Plac8	placenta-specific 8	231507	Liver 24	7.49
Bst2	bone marrow stromal cell antigen 2	69550	Lung 24	7.19
Herc6	hect domain and RLD 6	67138	PBMC 24	7.11
Rtp4	receptor transporter protein 4	67775	Kidney 24	7.06
Ifitm3	interferon induced transmembrane protein 3	66141	Heart 24	6.84
Tap1	transporter 1, ATP-binding cassette, sub-family B (MDR/TAP)	21354	Lung 24	6.83
Tap2	transporter 2, ATP-binding cassette, sub-family B (MDR/TAP)	21355	Kidney 24	6.61
Dhx58	DEXH (Asp-Glu-X-His) box polypeptide 58	80861	PBMC 24	6.36
Ifi204	interferon activated gene 204	15951	PBMC 24	6.19
H28	histocompatibility 28	15061	Lung 24	6.15
Parp9	poly (ADP-ribose) polymerase family, member 9	80285	Kidney 24	6.03
Cmpk2	cytidine monophosphate (UMP-CMP) kinase 2, mitochondrial	22169	PBMC 24	6.01
Xaf1	XIAP associated factor 1	327959	Liver 24	5.82
Irf1	interferon regulatory factor 1	16362	PBMC 5	5.79
Parp14	poly (ADP-ribose) polymerase family, member 14	547253	Kidney 24	5.67
Mlkl	mixed lineage kinase domain-like	74568	PBMC 24	5.65
Parp12	poly (ADP-ribose) polymerase family, member 12	243771	PBMC 24	5.65
Ly6e	lymphocyte antigen 6 complex, locus E	17069	Heart 24	5.48
Tapbpl	TAP binding protein-like	213233	Heart 24	5.39
Trim30a	tripartite motif-containing 30A	20128	Lung 24	5.10
Apol9a; Apol9b	apolipoprotein L 9a; apolipoprotein L 9b	223672; 71898	Lung 24	5.00
Oas1a	2′-5′ oligoadenylate synthetase 1A	246730	Lung 24	4.92
Tapbp	TAP binding protein	21356	Kidney 24	4.92
Lgals3bp	lectin, galactoside-binding, soluble, 3 binding protein	19039	Kidney 24	4.75
Gm9706^b^	predicted gene 9706	677168	PBMC 24	4.50
Dtx3l	deltex 3-like (Drosophila)	209200	Lung 24	4.41
Ifi35	interferon-induced protein 35	70110	Kidney 24	4.41
Nmi	N-myc (and STAT) interactor	64685	Kidney 24	4.26
Rnf213	ring finger protein 213	672511	PBMC 24	4.26
Ly6c1; Ly6c2	lymphocyte antigen 6 complex, locus C1; locus C2	17067; 100041546	Liver 24	4.17
H2-T10; H2-T22; H2-T9	histocompatibility 2, T region locus 10; locus 22; locus 9	15024; 15039; 15051	Heart 24	4.16
Uba7	ubiquitin-like modifier activating enzyme 7	74153	Heart 24	4.14
Psme2	proteasome (prosome, macropain) 28 subunit, beta	19188	Kidney 24	4.04
Ifih1	interferon induced with helicase C domain 1	71586	PBMC 24	3.91
Tnfsf10	tumor necrosis factor (ligand) superfamily, member 10	22035	Heart 24	3.85
Samhd1	SAM domain and HD domain, 1	56045	Heart 24	3.68
Erap1	endoplasmic reticulum aminopeptidase 1	80898	Kidney 24	3.66
Trafd1	TRAF type zinc finger domain containing 1	231712	PBMC 24	3.16
Psme1	proteasome (prosome, macropain) 28 subunit, alpha	19186	Kidney 24	2.91
Irf8	interferon regulatory factor 8	15900	Lung 24	2.72
Eif2ak2	eukaryotic translation initiation factor 2-alpha kinase 2	19106	Lung 24	2.48
Trim12c; Trim5	tripartite motif-containing 12C; tripartite motif-containing 5	319236; 667823	Kidney 24	2.46
Vwa5a	von Willebrand factor A domain containing 5A	67776	PBMC 24	2.02

Genes are ordered by maximum fold change from baseline. For two or more probesets annotated to the exact same gene and Entrez ID, results are only shown for the probeset with the largest fold-change. Three probable duplicate entries, with tentative annotations and more than one Entrez ID number, are designated (a, b, c, respectively), leaving a total of 82 unique genes. Tissue and time in hours of maximum fold changes are shown. Multiple probesets for the same gene occasionally showed more than two fold differences in maximum fold-change, but did not differ by tissue or time point of peak effects.

### Pattern and magnitude of the all-tissue shared response

**Figure 1:**
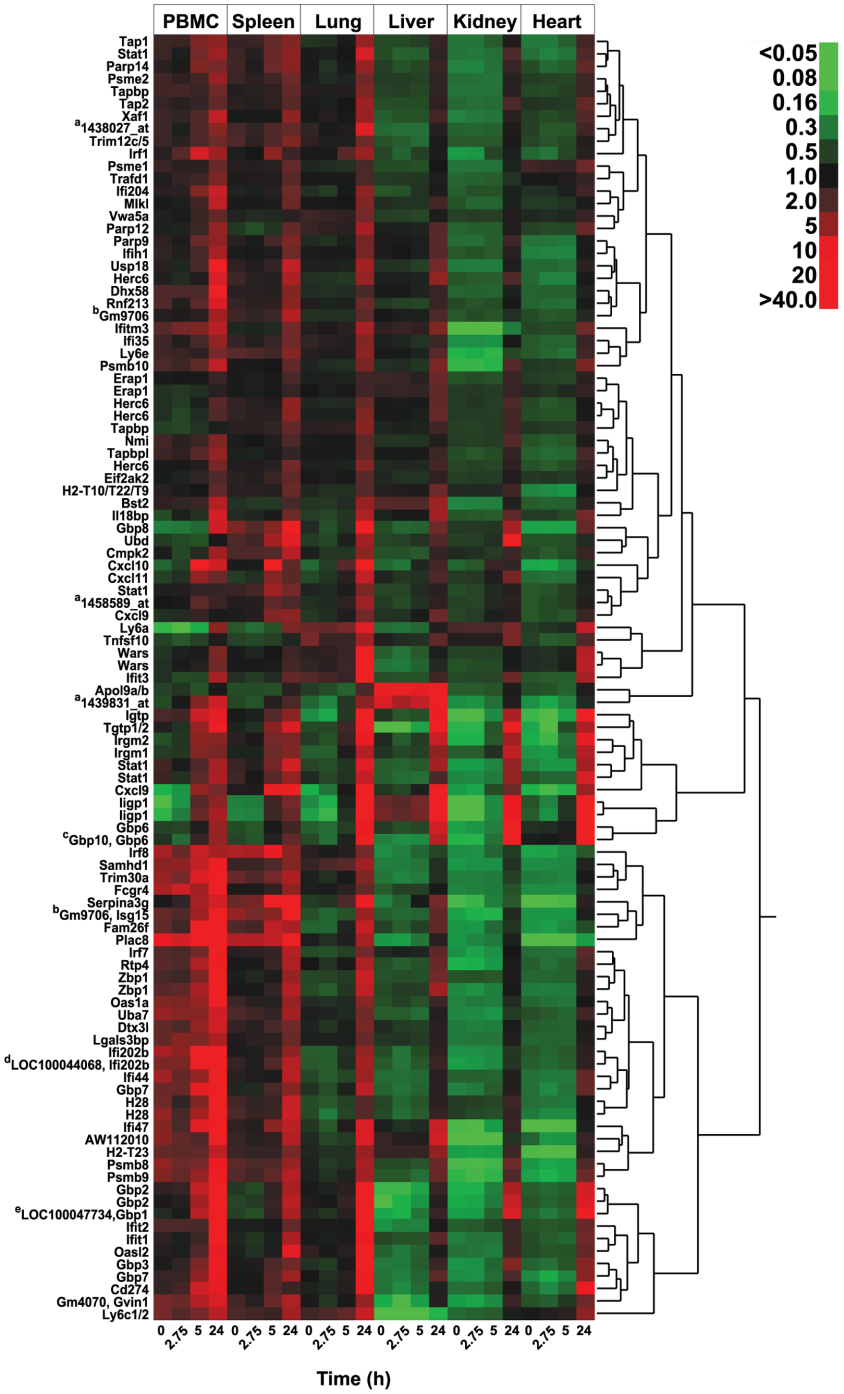
Heatmap of 103 probesets differentially regulated in all tissues. Probesets (≤5% FDR; ≥1.5-fold-change compared to control; and ≥50% present call within at least one condition/time point, across all tissues) are displayed on the vertical axis and designated, when available, by the symbol of the gene to which each is annotated, including duplicates. Tissue and time points are denoted on the horizontal axis. Each probeset has been normalized to its mean value across all times and tissues within one row. Red signifies expression above and green below the mean value within an individual row. As shown, baseline expression of these differentially expressed transcripts tends to decrease from PMBC > Spleen > Lung > Liver > Kidney, Heart. In contrast, all of these genes are induced by staphylococcal enterotoxin B (SEB) challenge with most reaching their highest levels of expression at 24 h across all tissues. ^a^Three unannotated probesets, identified only by Affymetrix® probeset IDs; ^b^Predicted gene Gm9706 of unknown function; second probeset annotated to Gm9706 is also annotated to the gene symbol Isg15; while these probesets do not cluster together, peak expression for both were seen in PBMCs at 24 h, suggesting that they may interrogate the same gene, but with different efficiencies; ^c^Probably detecting Gbp6 with which it clusters, but this probeset retains its annotation to both Gbp10 and Gbp6 as shown; ^d^Probably detecting Ifi202b with which it clusters, but this probeset retains its annotation to both LOC100044068 and Ifi202b as shown; ^e^Probably detecting Gbp1, but this probeset retains its annotation to both LOC100047734 and Gbp1 as shown.

A heatmap of the 103 probesets participating in this shared-response across tissues is shown in Figure 1. Gene symbols are shown when available; duplicate labels denote that more than one probeset was annotated to the same transcript. As shown, all-tissue shared-response genes tended to be more highly expressed (red) in immune tissue (PBMCs and spleen) at baseline. Furthermore, responses as early as 5 h post-challenge are only apparent on this heatmap in PBMCs and spleen, and involve relatively few leading-edge transcripts. Otherwise, the shared genomic response to SEB was highly consistent across all-tissues and most apparent at 24 h. Notably, these changes in gene expression precede the first deaths in this model by more than 48 h [31]; 24 h was chosen for study here because it is the latest known time point at which initiation of immune modulator therapy still affords protection [43].

**Figure 2:**
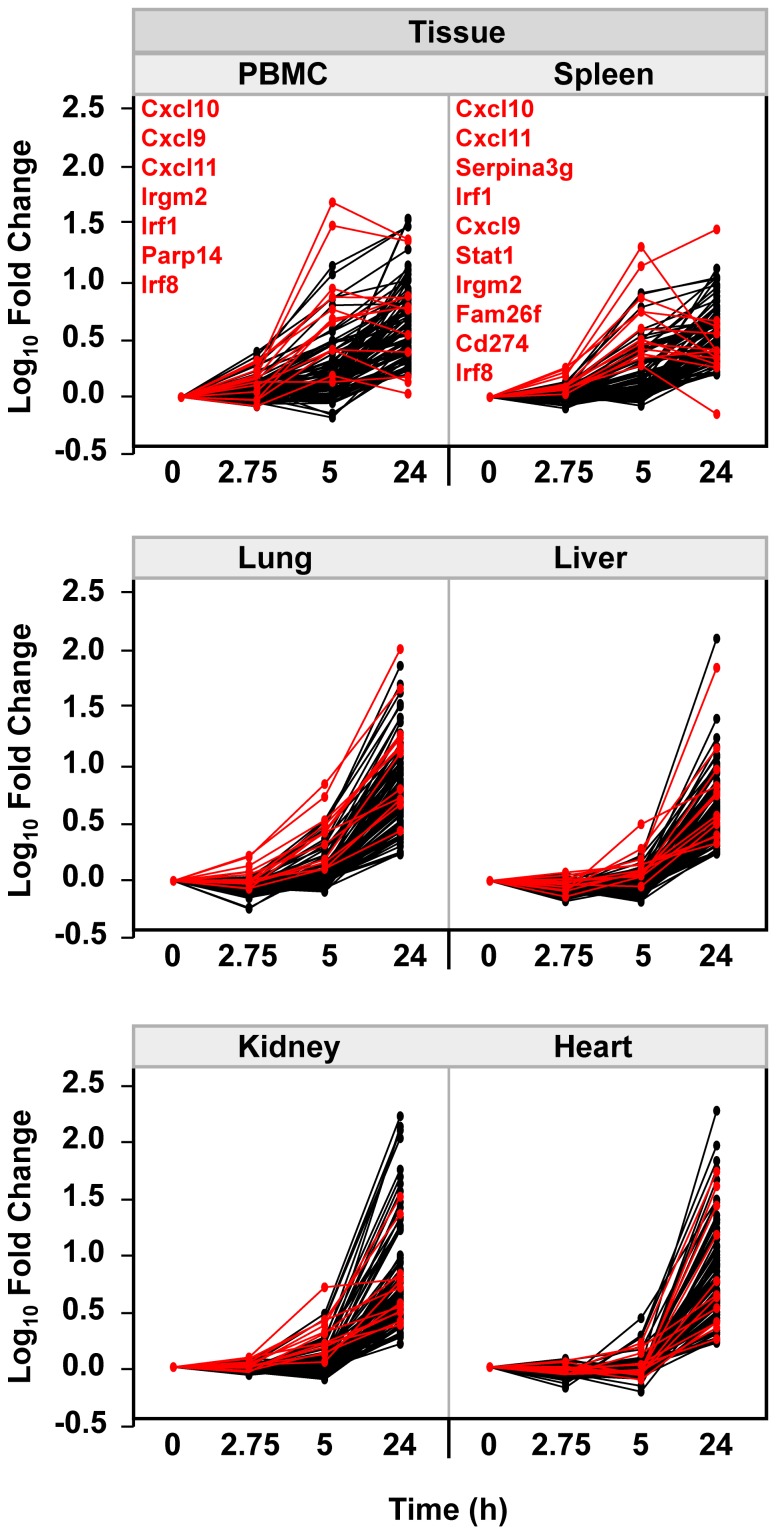
Tissue-specific parallel plots of the 103 probesets that met selection criteria. Expression levels were normalized to the time 0 h control condition to emphasize change over time from baseline. Probesets with peak expression before 24 h in any tissue are displayed in red. Notably, 12 probesets representing 11 unique genes (Cxcl9, Cxcl10, Cxcl11, Cd274, Fam26f, Irf1, Irf8, Irgm2, Parp14, Serpina3g, and Stat1) peaked at 5 h post-staphylococcal enterotoxin B (SEB) challenge in PBMCs and/or spleen as indicated. Gene symbols (in red) are displayed vertically from highest to lowest fold-change at 5 h.

To better illustrate the commonality of this shared-response in PBMCs and across multiple organs, parallel plots were generated within tissue, normalizing each of the 103 probesets to its own 0 h baseline expression level (Fig. 2). As seen, expression changes were generally maximal at 24 h in all tissues. However, 12 probesets (shown in red) representing 11 leading-edge transcripts (Cxcl9, Cxcl10, Cxcl11, Cd274, Fam26f, Irf1, Irf8, Irgm2, Parp14, Serpina3g, Stat1) reached their peak response in PBMCs and/or spleen at 5 h post-SEB challenge. Notably, 3 of these 11 genes are transcription factors (Irf1, Irf8, and Stat1) downstream from traditional IFN signaling, and 3 are recognized as early/immediate IFN-induced chemokines (Cxcl9, Cxcl10, and Cxcl11). These results suggest that an IFN-type response might have been initiated in PBMCs and spleen with later generalization to other tissues.

**Figure 3:**
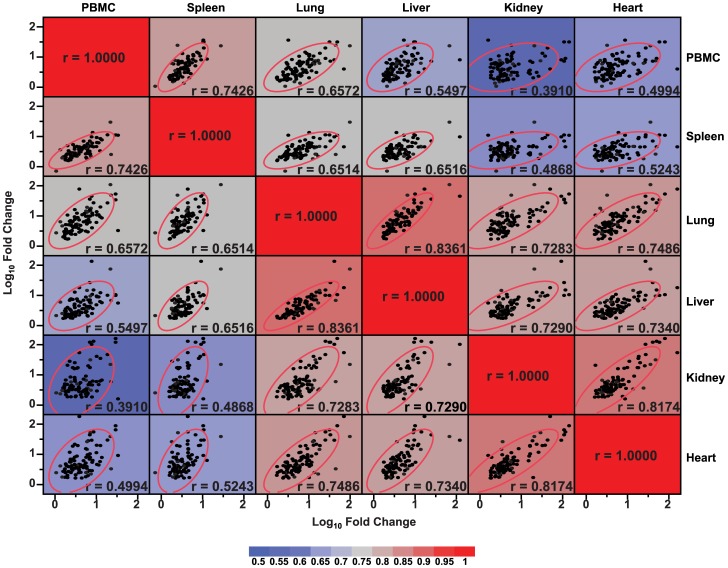
Correlation matrix of gene expression levels by tissue type. Expression levels of 103 probesets are shown as log_10_ fold-change relative to control (saline-exposed) animals at 24 h after staphylococcal enterotoxin B (SEB) challenge. Tissue-to-tissue comparisons using Pearson's correlation are represented numerically by r-values and in shades of red (higher correlation) and blue (lower correlation). Tissue/organ pairs with the closest patterns of gene expression were PBMC/spleen, lung/liver, and kidney/heart.

Finally, this coordinated genomic response was examined in a correlation matrix to determine which tissues displayed the most similarity in expression patterns at 24 h (Fig. 3). Comparisons with the highest correlation coefficients are shown in red and the lowest are in blue. Not unexpectedly, the two tissues comprised largely of lymphocytes, PBMCs and spleen, correlated closely with each other (r = 0.74). Interestingly, lung displayed a response most similar to liver (r = 0.84), possibly reflecting the large number of tissue macrophages in both organs or that SEB challenges were delivered into the bronchial tree and peritoneal cavity, respectively. Perhaps least anticipated were the shared-responses at 24 h in kidney and heart (r = 0.82; Fig. 3). These anatomically, histologically, and functionally dissimilar organs were both relatively distant from the sites of SEB challenge. Nonetheless, maximum fold-changes for a substantial number of transcripts occurred in these two organs (Fig 3 and Table 3).

### Quantitative real-time PCR (qRT-PCR) confirmation of microarray results

**Figure 4:**
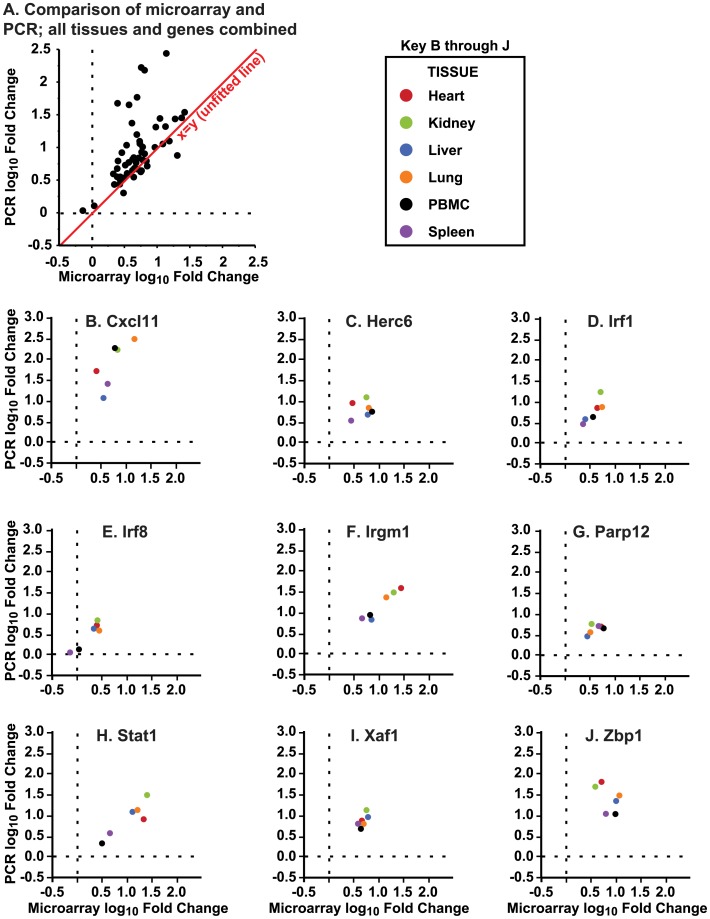
Quantitative real-time PCR (qRT-PCR) confirmation of tissue-wide changes in gene expression. Nine genes were quantitated by qRT-PCR across all 6 tissues at 24 h. (A) Scatter plot of all genes and tissues tested comparing microarray and qRT-PCR fold-change from control. As shown by the line of identify (x  =  y), qRT-PCR typically returned higher fold-change results than microarray. Gene specific results, colored by tissue (see Legend), are shown as follows: (B) Cxcl11; (C) Herc6; (D) Irf1; (E) Irf8; (F) Irgm1; (G) Parp12; (H) Stat1; (I) Xaf1; and (J) Zbp1. All qRT-PCR results met the >1.5 fold-change cut-off for gene selection, except for measurements of Irf8 in PBMCs and spleen. However, Irf8 similarly failed selection by microarray in these tissues at 24 h. Four samples were tested per tissue. Each PBMC sample represented a pool of multiple mice while each organ sample came from an individual mouse.

Nine genes differentially regulated across all tissues (Cxcl11, Herc6, Irf1, Irf8, Irgm1, Parp12, Stat1, Xaf1, Zbp1) were selected for validation by qRT-PCR. Each gene was tested in 4 separate samples from each of 6 tissues obtained at 24 h after SEB challenge. Microarray tended to underestimate fold-change from control compared to qRT-PCR results, as shown by the systematic deviation from the line of identity in Fig. 4A. Overall, qRT-PCR confirmed that every differentially regulated gene was induced >1.5 fold compared to control in all six tissues (Fig 4B to 4J), with the exception of Irf8 (Fig.4E). Irf8 had met inclusion on the all-tissue list by passing selection criteria at 5 h in PBMCs and spleen and at 24 h in the 4 other tissues. As such, qRT-PCR and microarray showed close agreement at 24 h; both methods demonstrated that Irf8 was only differentially expressed in lung, liver, kidney and heart at this time point.

### Thematic and functional analysis

**Figure 5:**
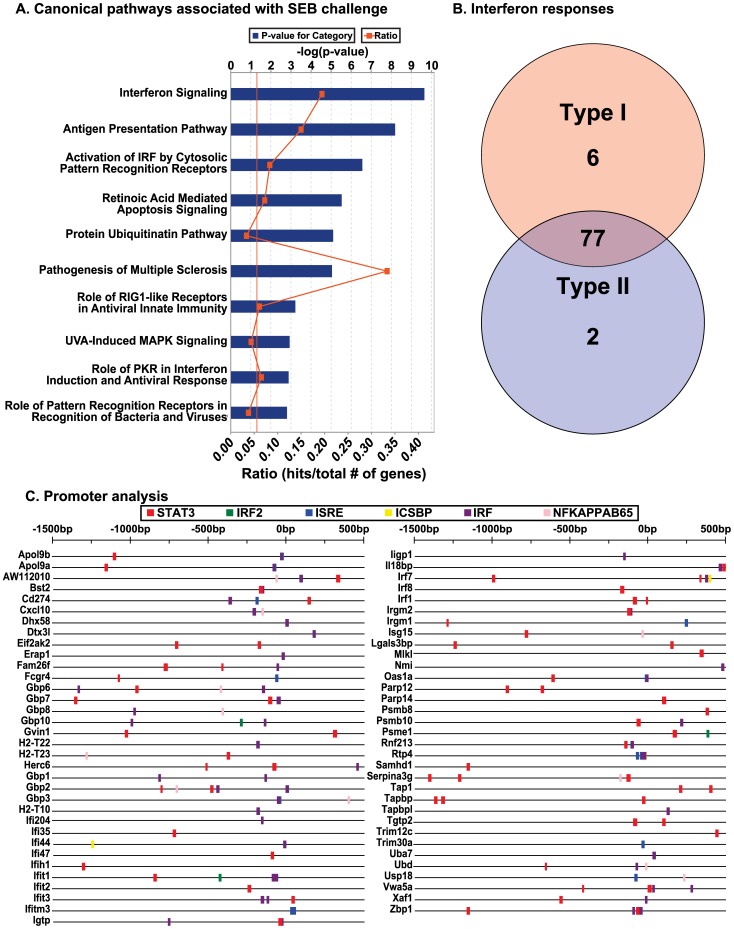
Thematic analysis, interferon (IFN) response subtype classification, and promoter analysis for binding matrices responsive to IFN. (A) Canonical pathways significantly associated with the all-tissue response to staphylococcal enterotoxin B (SEB) challenge. Seventy-nine unique genes were recognized by the Ingenuity Pathway Analysis® (IPA®) database and mapped to IFN signaling, antigen presentation, and activation of IFN regulatory factor (IRF) by cytosolic pattern recognition receptors, among the other canonical pathways shown. (B) Classification of genes significantly up-regulated across all tissues by IFN response subtype. Note that for *Mus musculus*, the Interferome v2.01 database contained 1655 Type I genes, 1413 Type II genes, and no Type III genes. (C) IFN pathway-driven regulatory binding sites identified in the promoters of genes regulated across all tissues. Of 81 promoter regions analyzed (from +500 to −1500 bp), 68 were found to contain IFN-driven regulatory matrices as shown. Results generated by Interferome v2.01 using TRANSFAC® Professional (2012) matrices and the MATCH™ algorithm.

Of 103 probesets uploaded into IPA®, 79 unique transcripts were recognized by the database. As simple inspection had already indicated, top functional categories for these genes included inflammatory response and antigen presentation. The all-tissue gene list was also significantly associated with liver, kidney and heart toxicity (data not shown). A canonical pathway analysis identified an IFN-biased innate immune response that seemed more appropriate for viral infection rather than a bacterial toxin (Fig. 5A). Top, highly significant canonical pathways included IFN signaling, antigen presentation, interferon regulatory factor (IRF) activation by cytosolic pattern recognition receptors, retinoic acid-mediated apoptosis signaling, protein ubiquination pathway, pathogenesis of multiple sclerosis, and role of retinoic acid-inducible gene (RIG)-like receptors in anti-viral immunity.

Because of the overwhelming IFN-response signature, the all-tissue gene list was next analyzed in a custom database (Interferome v2.0) of curated IFN-regulated genes from publically available microarray experiments [42]. Using mouse-specific IFN-regulated gene lists and default 2.0 fold-change cutoff values, 85 uniquely annotated transcripts were classified as belonging to type I, type II, or both type I and II, IFN-response subtypes (Fig. 5B). Next, the promoters of these genes were analyzed within the Interferome v2.0 environment for the presence of IFN-responsive regulatory elements. Of 81 available promoter sequences, 68 returned one or more promoter matrices associated with IFN regulation (Fig 5C).

To further test whether IFN-regulated binding sites were truly over represented among genes up-regulated by SEB across all tissues in our animal model, these promoters were next compared to a large set of mouse housekeeping genes (BIOBASE Knowledge Library®) using an all vertebrate promoter matrix profile from TRANSFAC® Professional (see Methods). Of 33 significantly enriched promoter matrices (matched promoter FDR<0.05), the top 11 ranked from lowest to highest FDR were all regulated by IFN activated and/or induced transcription factors (Table 4). Note in Table 4 that Prdm1 (Blimp1), a Irf4-regulated repressor essential for B-cell, T-cell and natural killer cell maturation, competitively interacts with IFN-stimulated response elements (ISREs) [44] and IRF binding sites [45] in target promoters.

**Table 4:** Top eleven promoter regulatory matrices identified using the F-match module in ExPlain 3.1 (BioBase Knowledge Library) (see Methods).

Promoter Matrix Name	Associated Transcription Factors	Yes (sites/1000 bp)	Yes/No^a^	From	To	Matched promoters FDR
V$ISRE_01	Irf7, Irf8, ISGF3G, STAT1, STAT2	0.2778	infinity	−500	100	4.6E-09
V$IRF7_01	Irf7, Irf8, ISGF3G, STAT1, STAT2	0.3373	infinity	−500	100	4.6E-09
V$IRF1_Q6_01	Irf1	0.2579	infinity	−400	100	9.2E-08
V$IRF_Q6_01	Irf1, Irf2, Irf3, Irf4, Irf5, Irf6, Irf7, Irf8, Isgf3g, STAT1, STAT2	0.2183	infinity	−400	100	5.3E-07
V$IRF_Q6	Irf1, Irf2, Irf3, Irf4, Irf5, Irf6, Irf7, Irf8, Isgf3g, STAT1, STAT2	0.1984	infinity	−400	100	2.4E-05
V$BLIMP1_Q6	Prdm1^b^	0.1786	infinity	−400	100	2.4E-05
V$IRF1_Q6	Irf1, Irf2	0.4365	6.78	−200	100	3.3E-05
V$ICSBP_Q6	Irf7, Irf8, ISGF3G, STAT1, STAT2	0.1786	infinity	−200	100	1.1E-04
V$IRF7_Q3	Irf7	0.7143	4.05	−200	100	6.3E-04
V$IRF8_Q6	Irf8	0.2778	9.11	−200	100	7.6E-04
V$IRF3	Irf3	0.1786	17.57	−500	100	1.2E-03

a Ratio of the abundance of each promoter matrix in genes differentially regulated across all six tissues compared to 492 mouse housekeeping genes (see Methods).

b Prdm1 (Blimp1), a transcriptional repressor essential for B- and T-cell differentiation and homeostasis, is regulated by Irf4. Prdm1 and interferon regulatory factors bind to similar DNA sequences. Some promoters contain overlapping motifs where Prdm1 and Irf family members may competitively interact.

Next, expression results from individual tissues were examined for secreted IFN pathway initiators, as possible up-stream sources of this tissue/organ-wide IFN response. Twenty-six candidate genes were identified from PubMed and other online databases, of which 19 mapped to the mouse oligonucleotide microarrays used in this study. Notably, none of these genes appeared on our all-tissue list of differentially regulated transcripts. However, 8/19 were found to be significantly up-regulated in PBMCs, spleen and/or lung prior to 24 h (Table 5), suggesting that the products of these genes might be contributing to the global IFN-type response seen here.

**Table 5:** Interferon pathway inducing genes (fold change, SEB *versus* control).

			Fold Change																	
			PMBC			Spleen			Lung			Liver			Kidney			Heart		
GeneID	Gene Title	Tissues meeting all criteria (5%FDR; 1.5FC)	2.75 h	5 h	24 h	2.75 h	5 h	24 h	2.75 h	5 h	24 h	2.75 h	5 h	24 h	2.75 h	5 h	24 h	2.75 h	5 h	24 h
15978	interferon gamma	Spleen, PBMC, Lung	0.98	1.18	***1.71***	***1.51***	***2.50***	1.27	1.17	***1.58***	***3.55***	1.02	0.92	***1.67***	1.07	1.17	1.18	1.00	0.88	1.02
16175	interleukin 1 alpha	PBMC	1.46	***6.24***	***1.56***	1.09	1.47	1.21	0.97	1.02	1.16	1.18	1.40	1.38	0.96	1.01	1.17	0.97	0.90	1.05
16176	interleukin 1 beta	Spleen, PBMC	0.75	***5.54***	***1.97***	1.44	***4.12***	***2.06***	0.55	***2.78***	***4.95***	1.04	***2.02***	1.45	0.95	1.23	1.19	0.85	1.09	1.09
16183	interleukin 2	Spleen, PBMC, Lung	***1.95***	***1.89***	1.08	***4.58***	***4.83***	0.98	1.37	***2.92***	1.29	1.20	1.45	0.99	1.01	0.97	1.16	1.04	0.89	1.15
16160	interleukin 12b	Spleen, Lung	1.17	1.30	1.23	1.29	***2.16***	0.82	1.06	1.03	***1.63***	1.09	1.13	1.24	1.01	1.21	1.16	1.01	1.33	1.15
21926	tumor necrosis factor	PBMC	***1.76***	***7.04***	***1.54***	***1.95***	***2.90***	1.24	1.20	1.46	***2.47***	1.03	1.05	0.90	1.02	1.03	1.10	1.00	0.97	0.91
12977	colony stimulating factor 1	PBMC	0.86	***1.96***	***1.89***	1.08	1.20	0.75	1.14	1.45	***1.81***	0.91	1.02	***1.80***	0.95	1.15	***1.84***	0.83	0.93	1.24
12985	colony stimulating factor 3	Lung	0.91	1.48	1.14	1.06	***2.32***	1.12	1.26	***1.73***	1.37	0.87	1.13	1.09	1.09	1.03	0.97	0.81	0.83	0.90

Of 26 genes identified as potential interferon pathway initiators, 19 were mapped to probesets on our microarray and 8 of these were significantly up-regulated (FDR 5% and 1.5 fold-change from control) in three tissues, PBMCs, spleen and/or lung. Tissues and time points meeting at least the 1.5 fold-change criteria are shown in bold italics.

**Figure 6:**
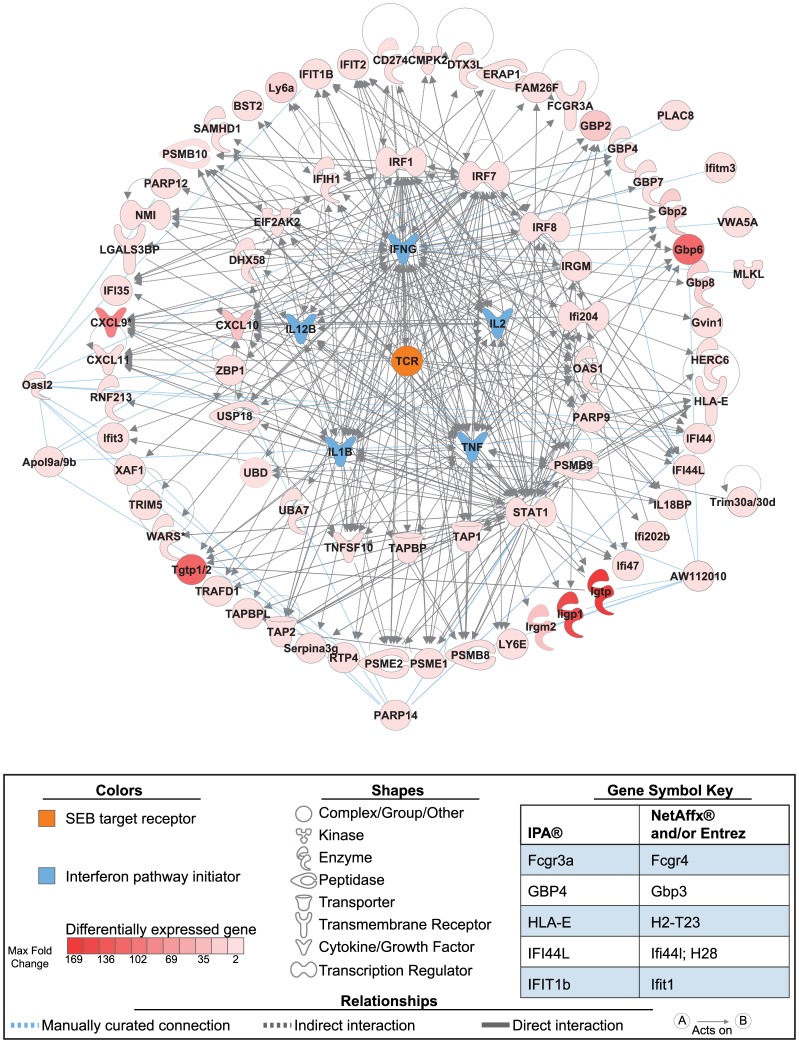
Functional network of selected upstream-regulators and differentially expressed genes across all tissues. From among the significant nodes identified using the Ingenuity Pathway Analysis® (IPA®) Upstream Regulator tool, the following were selected for inclusion in the displayed network: 1) the T-cell receptor (TCR), as this is the primary target of staphylococcal enterotoxin B (SEB)-mediated cell activation (colored orange at the network center); 2) TNF, IL-1β, IL-2, IFNγ and IL-12B, as these are known interferon (IFN) pathway initiators that were expressed early in the peripheral blood mononuclear cells and/or spleens of the SEB challenged mice (colored blue and positioned as the inner most ring of the network); and 3) any upstream regulator that was also present on our all-tissue list of differentially expressed genes (colored in shades of red proportional to fold-change) and positioned as the next ring moving outward. The resulting network connected 70 of 79 genes recognized by IPA®. The remaining 9 genes (outside of the outermost ring) were connected manually (see text) using PubMed and STRING (http://string-db.org/newstring_cgi/) version 9.05, a database of known and predicted protein-protein interactions. A key defining colors, shapes, and relationships is shown. In addition, changes in gene symbols from those in Figure 1 and Table 3 are provided for clarity. Also note that IPA® frequently defaults to all-capital gene symbols that denote human genes, while elsewhere the mouse format is followed of only capitalizing the first letter.

Finally, the Upstream Regulators application in IPA® was used to construct a network of gene interactions that encompassed activation of TCRs by SEB and the IFN pathway activating molecules identified above (Fig. 6). Notably, of 79 genes recognized by the database, 70 were connected by the program into a functional network of genetic and protein-protein interactions. The remaining nine genes were then added to this network based on a review of direct experimental evidence manually curated from PubMed and other online resources (see Methods).

### Pathology and immunohistochemistry

**Figure 7:**
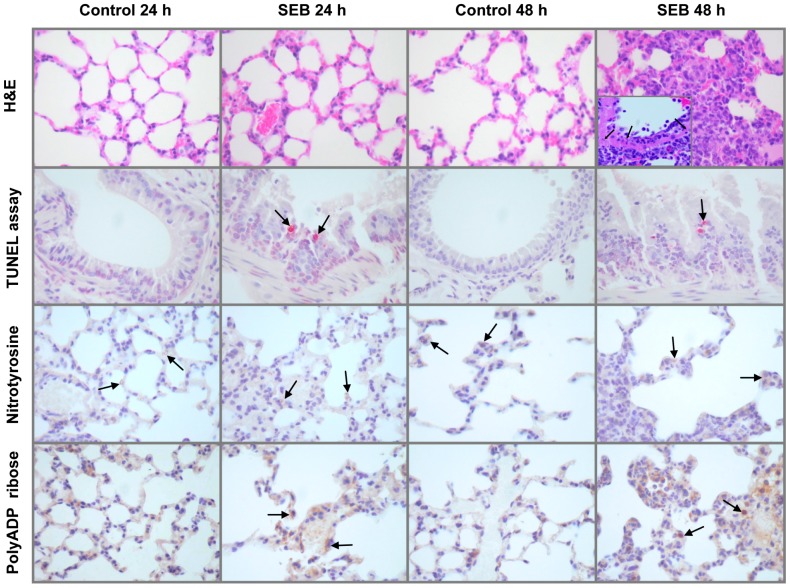
Pulmonary pathology: hematoxylin and eosin (H&E) stain, TUNEL assay and immunohistochemistry staining for nitrotyrosine and polyADP-ribose. Compared to control animals at 24 h, staphylococcal enterotoxin B (SEB) challenge caused a multifocal, minimal to mild perivascular, peribronchiolar, interstitial and subpleural lymphohistiocytic inflammatory infiltrate. At 48 h a coalescing, neutrophil-predominant infiltrate was seen in SEB exposed animals that now extended into alveoli. Multiple vessel walls 48 h after SEB exposure contained neutrophilic fragments (arrows) consistent with vasculitis (H&E inset, SEB 48 h). Terminal deoxynucleotidyl transferase-mediated dUTP nick end labeling (TUNEL) assay demonstrated an increase in bronchiolar apoptotic cells (arrows) after SEB challenge compared to control that was significant at 24 h post-exposure (2.93±0.12 *versus* 0.06±0.06 cells/HPF; *p<0.001*). Immunohistochemistry for nitrotyrosine was not different comparing SEB to control with all specimens showing faint staining (arrows) of alveolar epithelium, small vessel endothelium and alveolar macrophages. In contrast, immunohistochemistry for polyADP-ribose (PAR) showed increased staining associated with SEB exposure that was mostly proportional to the increase in inflammatory cellularity. At 48 h, hypertrophied alveolar epithelial cells (arrows) stained prominently for PAR.

At 24 h comparing SEB-challenged animals to controls, inflammatory cellular infiltrates were only seen in lung tissue. Therefore, gene expression changes in liver, kidney and heart 24 h after SEB exposure were not due simply to the influx of immune cells. In lung tissue at 24 h, SEB-challenge caused a multifocal minimal to mild perivascular, peribronchiolar, interstitial and subpleural lymphohistiocytic inflammatory infiltrate (Fig. 7). A coalescing, neutrophil-predominant infiltrate was seen in SEB exposed animals by 48 h that extended into alveolar spaces (Fig. 7). Furthermore, the walls of some small vessels at 48 h contained neutrophilic fragments consistent with vasculitis (Fig. 7 inset). Because these SEB-associated changes could serve as a quality control measure, lung tissue was chosen to further study apoptosis, free radical injury, and polyADP ribosylation.

SEB challenge compared to control produced a significant increase (2.93±0.12 *versus* 0.06±0.06 cells/HPF; *p<0.001*) in apoptotic cells associated with bronchioles as measured by terminal deoxynucleotidyl transferase-mediated dUTP nick end labeling (TUNEL) assay (Fig. 7; arrows). Nitrotyrosine staining, a measure of peroxynitrite-mediated oxidant injury, did not differ between the two groups with all specimens showing similar amounts of faint staining (Fig. 7; arrows) in alveolar epithelium, small vessel endothelium and alveolar macrophages. In contrast, immunohistochemistry staining for polyADP-ribose (PAR), a product of poly [ADP-ribose] polymerase (PARP) enzymatic activity, showed increased staining associated with SEB exposure that was largely cytosolic and proportional to the increase in inflammatory cellularity. At 48 h, hypertrophied alveolar epithelial cells (Fig. 7; arrows) stained prominently for PAR. Notably, three macro-PARP genes, Parp9, Parp12 and Parp14, were significantly up-regulated across all tissues in mice challenged with a lethal dose of SEB.

## Discussion

Oligonucleotide microarrays were used to analyze the global, host-wide response to SEB in a murine model of superantigen-mediated shock that uses relatively low-dose challenges without the need for priming agents and does not cause rapid death [31]. Although relevance to human disease is not certain, our finding of a late type I/II IFN response across multiple organs and tissues lends support to the possible importance of this pathway in toxic shock syndromes. Eighty-five genes that annotated to an IFN antiviral response were uniformly up-regulated in PBMC, spleen, lung, liver, kidney, and heart. Potential initiators of IFN signaling such as IL-2, IL-12B and IFNγ were only modestly up-regulated at early time points in some tissues, while IFN regulatory factors and Zbp1, a DNA sensor/transcription factor that directly elicits IFN innate responses, were notable components of the host-wide SEB signature. These results suggest that therapies aimed at IFN-associated innate immune responses may improve outcome in human toxic shock.

Despite important clinical distinctions between staphylococcal menstrual [1] and non-menstrual [46], [47], [48], and streptococcal [2] forms of toxic shock, the various exotoxins associated with each of these syndromes including SEB share a common mechanism that leads to intense activation of the host immune system. In each case, the putative exotoxin binds directly to TCRs on T-cells and to major histocompatibility complex class II molecules on antigen-presenting cells, forming a bridge [5]. Co-stimulatory receptors on both cells, such as CD28 and CD80, also bind to each other triggering a massive polyclonal inflammatory response [11], [12]. The ensuing cytokine storm with the rapid release of TNFα, IL-1β, IL-2 and IFNγ has been generally held responsible for all of the subsequent, clinical consequences of toxin exposure including hypotension, multiple organ injury and death [6], [21], [22]. Here, a shared genomic response across all tissues and organs was found that annotated to 85 up-regulated genes. While this shared response represented only a small fraction of all differentially expressed transcripts in PBMCs (3%), its dominance as a proportion of involved genes grew from spleen and lung (11% each) to liver, kidney and heart (all>30%) and over time. The simultaneous, concordant expression of transcripts across multiple organs suggested a common transcriptional regulatory mechanism that might be central to pathogenesis and possibly provide insights into treatment. Ultimately, all of these shared genes could be mapped to a dominant type I and type II IFN-signature. Notably, other investigators using animal models [26], [49], [50], [51] or studying the cytokine response in patients with toxic shock syndrome [22] have provided evidence for the possible importance of IFN signaling in the pathogenesis of superantigen-mediated disease. Recently, Tilahun et al found that Ifnγ knockout conferred significant protection from lethal SEB challenge in a HLA-DR3 transgenic mouse model of toxic shock syndrome [51]. Collectively with previous work, our results suggest that IFN targeted therapeutic approaches warrant investigation.

The molecular mechanisms that drive this host-wide IFN-response 24 h after SEB exposure are not clear, but several possibilities are suggested by the list of affected genes. While, IFNα/β was not up-regulated at any time point in any tissue, other IFN pathway inducers, including IL-2, IL-12B, and IFNγ, were induced early, but only modestly so, in PBMCs, spleen and lung, and may have contributed to the shared IFN-response. Also supporting this notion, a number of the transcripts up-regulated across all tissues are known to amplify IFN regulated gene transcription. PARP9 and PARP14 remodel chromatin and NMI interacts with STAT (signal transducer and activator of transcription) proteins to increase transcriptional responses to IL-2 and IFNγ [52], [53], [54].

An alternative explanation for the general IFN-response found in our model is suggested by the all tissue induction of Zbp1, a transcription factor activated by cytosolic, double-stranded DNA fragments, whether microbe- or host-derived, that can induce type I IFN genes independent of IFNα/β signaling [55], [56]. Several poly [ADP-ribose] polymerases (Parp9, Parp12 and Parp14) and Dtx3l, an E3 ubiquitin ligase co-regulated with Parp9 through a shared promoter, were also up-regulated in every tissue tested and functionally annotate to DNA repair [57], [58]. Together the expression of Zbp1, a DNA sensor, and multiple DNA damage/repair genes suggests that the host-wide IFN-signature reported here might be driven by multi-organ cellular injury. Nuclear or mitochondrial DNA leakage into the cytoplasm of injured cells could trigger damage[danger]-associated molecular pattern (DAMP) recognition, resulting in an IFN-response [59], [60], [61]. However, some caution is warranted in drawing this conclusion. PARP12 has recently been recognized as a cytoplasmic, post-transcriptional regulator with antiviral activity [62], [63] and may not function primarily in DNA damage/repair. Importantly, immunohistochemical staining for poly-ADP-ribose (PAR) was largely cytosolic and widespread tissue damage was not seen by histopathology at 24 h post-exposure.

Epigenetic remodeling may also serve to activate IFN-type antiviral gene responses independent of or at least additive with IFNα/β/γ signaling in non-immune cells. Fang and colleagues found that blocking histone 3 lysine 9 di-methylation (H3K9me2) led to a robust IFN signature and viral resistance in fibroblasts [64]. Notably, the IFN-regulated genes that were restricted by H3K9me2 overlap extensively with our all-tissue list. Furthermore, H3K9me2 epigenetic marks in neurons, cardiac myocytes, and other parenchymal cells are thought to suppress IFN responses and protect diverse cell types from IFN-induced tissue injury [64], [65].

The antiviral effector molecules induced by SEB in every organ tested included Dhx58, Eif2ak2, Herc6, Ifih1 (Mda5), Isg15, Oas1a, Oasl2, and Samhd1. DHX58 binds viral RNA and regulates Rig1 (retinoic acid-inducible gene 1), an intracellular pattern recognition receptor and viral sensor [66]. Likewise, IFIH1 (MDA5) is a RIG1-like receptor (RLR) family member that participates in viral defense [67]. HERC6 was recently identified as the main E3 ligase that catalyzes ISG15 conjugation (ISGylation) of proteins in mice to restrict the replication of a wide variety of viruses [68]. EIF2AK2 is activated by double-stranded RNA to block protein synthesis [69]. OAS1A and OASl2 are 2–5A synthetase family members that also bind double-stranded RNA and activate latent ribonuclease L, which degrades viral RNA [70]. Finally, SAMHD1 depletes the pool of nucleotides available to viral reverse transcriptases and thus prevents replication of HIV and other viruses [71]. Although latent virus activation cannot be entirely ruled out in our murine model of SEB challenge, viral cytopathic effects have not been seen on histopathology [31]. Nonetheless, further studies are needed to better understand the implications of this previously unrecognized, broad SEB-associated antiviral signature. At least superficially, this antiviral response would seem to be maladaptive in the setting of a bacterial infection with superantigen production.

Another somewhat surprising genetic signature found in our host-wide, shared response was the induction of immunoproteasome components (Psme2, Psmb8, Psmb9, and Psmb10) along with two ubiquitin ligases (Uba7 and Ubd), a deubiquitinating protease (Usp18), and Erap1, an aminopeptidase involved in antigen processing [72]. The protein products of these genes are integral parts in the machinery needed for the proper function of dendritic cells and other professional APCs [73]. Notably, UBD is known to promote the expression of the immunoproteasome component Psmb9, and plays an important role in dendritic cell maturation [74]. The induction of these genes might be anticipated in PBMCs and spleen. Likewise, lung and liver tissue have substantial numbers of macrophages and resident dendritic cells. In contrast, the strong expression of these genes in the kidneys and heart has not been reported previously. However, numerous studies have demonstrated that non-professional, antigen presenting-like cells can arise in non-myeloid cells of various tissues and organs, an occurrence that has been associated with autoimmunity [75]. Cells from the fibrous cap of human atherosclerotic lesions express the immunoproteasome component PSMB8 in response to IFNγ sensitization [76]. Finally, human endothelial cells exposed to either TSST-1 or SEB have been shown to express class II MHC molecules and to function as competent superantigen-presenting cells, possibly contributing to the vascular injury seen in patients with toxic shock syndrome [77].

The potential relevance of the shared, host-wide genomic program reported here to toxic shock syndrome is an important question. Recently, chronic infusion of low dose SEB in HLA-DQ8 transgenic mice was shown to produce a lupus-like syndrome involving multiple organs (lung, liver and kidney) [49]. STAT4 or IFNγ deficiency prevented this autoimmune-like tissue injury, supporting a pathogenic role for the Th1-type cytokines, IL-12 and IFNγ, in this model. More directly related to toxic shock and perhaps septic shock syndrome, mice with knockout of interferon-alpha receptor-1 (Ifnar1), and thus incapable of responding to type I IFNs (IFNα/β), were remarkably resistant to TNFα-induced inflammatory shock and death [78]. Importantly, IFNAR1 deficient mice were also protected from lethal *S. aureus* pneumonia compared to their wild-type counterparts [79]. This is notable as most immune system gene-knockouts are less fit to challenge with viable infectious agents. Collectively, these findings suggest that type I IFN responses, crucial in viral and intracellular pathogen defense, may be quite harmful in certain bacterial infections, at least in mice. Whether the late, host-wide, IFN-signature seen here is similarly detrimental and therefore a viable therapeutic target requires further investigation.

Dexamethasone and rapamycin have both been beneficial in our murine model of SEB lethality [43], [80]. Given very early (within 2 h) after SEB-challenge and then continued for a full 96 h, dexamethasone rescued animals from death and inhibited many inflammatory mediators, including several IFN-pathway initiators [80]. However, dexamethasone does not improve survival when given more than 5 h after SEB challenge. Whether late (after 5 h) administration of dexamethasone also fails to quell the host-wide IFN-signature reported here is not known. Unlike dexamethasone, very high-dose rapamycin prevented deaths, even when given as late as 24 h after SEB [43], the last time point we analyzed by microarray. The mammalian target of rapamycin (mTOR) has very complex effects on immunity. Inhibition of mTOR by rapamycin decreases T-cell proliferation and suppresses type I IFN responses [81], but can increase monocyte/macrophage-mediated inflammation [82], [83]. Lethality and IL-1β levels were increased by rapamycin in LPS-challenged mice, probably because mTOR suppression of NFκB and caspase-1 was blocked [84]. Interestingly, several genes on our all-tissue list were blocked by rapamycin in CpG oligodeoxynucleotides/TLR9-activated dendritic cells including Cxcl9, Gbp7, Ifit1, Igtp, Oasl1, and Rtp4 [84]. Overall, the beneficial effects of dexamethasone and rapamycin in our lethal mouse model of SEB-challenge [43], [80] support the potential pathogenic importance of the host-wide IFN-signature found here, but this hypothesis requires further testing.

In summary, a host-wide, innate IFN-response was seen across all tissues and organs in a lethal mouse model of SEB challenge. Whether this unexpected shared genomic program is primarily driven by the induction of IFN pathway inducers and amplifiers, DNA damage, and/or the recruitment of non-professional APCs into the generalized inflammatory response requires further study. Nonetheless, this multi-organ response to SEB exposure may contribute to the pathophysiology of SEB-induced shock and provides a rational for the specific interruption of these pathways to reduce inflammation and tissue injury. Because of the common mechanism of immune activation by all superantigens, therapeutics based on these findings could also be useful for the management of *de novo* toxic shock syndrome. Importantly, the generalized response seen here, characterized by IFN-inducible transcripts and intracellular sensors, unfolds in a manner such that an adequate window of time may exist for interventions based on this new view of superantigen lethality.

## Supporting Information

Table S1 **All probesets in any tissue meeting selection criteria (≤5% FDR; ≥1.5-fold-change compared to control; and ≥50% present call in at least one condition/time point).**
(XLSX)
